# Function of a viral genome packaging motor from bacteriophage T4 is insensitive to DNA sequence

**DOI:** 10.1093/nar/gkaa875

**Published:** 2020-10-29

**Authors:** Youbin Mo, Nicholas Keller, Damian delToro, Neeti Ananthaswamy, Stephen C Harvey, Venigalla B Rao, Douglas E Smith

**Affiliations:** Department of Physics, University of California, San Diego, La Jolla, CA 92093, USA; Department of Physics, University of California, San Diego, La Jolla, CA 92093, USA; Department of Physics, University of California, San Diego, La Jolla, CA 92093, USA; Department of Biology, The Catholic University of America, District of Columbia, 20064, USA; Department of Biochemistry and Biophysics, Univ. of Pennsylvania, Philadelphia, PA 19104, USA; Department of Biology, The Catholic University of America, District of Columbia, 20064, USA; Department of Physics, University of California, San Diego, La Jolla, CA 92093, USA

## Abstract

Many viruses employ ATP-powered motors during assembly to translocate DNA into procapsid shells. Previous reports raise the question if motor function is modulated by substrate DNA sequence: (i) the phage T4 motor exhibits large translocation rate fluctuations and pauses and slips; (ii) evidence suggests that the phage phi29 motor contacts DNA bases during translocation; and (iii) one theoretical model, the ‘B-A scrunchworm’, predicts that ‘A-philic’ sequences that transition more easily to A-form would alter motor function. Here, we use single-molecule optical tweezers measurements to compare translocation of phage, plasmid, and synthetic A-philic, GC rich sequences by the T4 motor. We observed no significant differences in motor velocities, even with A-philic sequences predicted to show higher translocation rate at high applied force. We also observed no significant changes in motor pausing and only modest changes in slipping. To more generally test for sequence dependence, we conducted correlation analyses across pairs of packaging events. No significant correlations in packaging rate, pausing or slipping versus sequence position were detected across repeated measurements with several different DNA sequences. These studies suggest that viral genome packaging is insensitive to DNA sequence and fluctuations in packaging motor velocity, pausing and slipping are primarily stochastic temporal events.

## INTRODUCTION

Most double-stranded DNA viruses, including many bacterial viruses (phages) and human/animal viruses including herpesviruses, poxviruses and adenoviruses, follow an assembly pathway in which the viral procapsid (prohead) shell assembles first and it is subsequently filled with DNA (1,2). An ATP-powered molecular motor translocates replicated viral DNAs into the procapsids via a portal nanochannel (2,3). DNA translocation by the phage phi29, lambda and T4 motors have been directly measured via single DNA molecule manipulation with optical tweezers (4–9). These studies revealed that viral motors are among the strongest known molecular motors, and able to exert forces of at least ∼60 pN (4–6). In comparison, a single skeletal muscle myosin II motor protein, also powered by ATP, exerts a maximum force of only ∼2–3 pN. More broadly, viral motors have been identified as members of an ASCE (additional strand, conserved E) superfamily of ATPases that play many critical roles in cell biology including nucleic acid unwinding, chromosome transport, and protein translocation and unfolding (10).

High force generation is a necessary property of the phage viral motors since high forces resist the tight confinement of the DNA genome, which is ultimately packed to near-crystalline density (2,11–12). Many theoretical and single molecule studies have investigated the physical factors that govern this process, including DNA bending and electrostatic self-repulsion, ionic-screening effects, entropy changes and nonequilibrium dynamics (13–21). The viral motors are sufficiently powerful that they can nevertheless translocate DNA rapidly against the opposing forces and package the viral genome in just a few minutes (22,23). A striking example is the phage T4 motor that was measured to translocate DNA at rates as high as ∼2000 bp/s and generate a power density as high as ∼5000 kW/m^3^ at room temperature with saturating ATP, which is twice that of a typical automobile engine (6).

The exact biochemical and structural mechanism by which viral motors function, and whether there is a universal packaging mechanism, is not fully understood although significant functional and structural information have been obtained on several model systems. Examples of recent progress, not intended to be a comprehensive review, include the following. Atomic-resolution structures of motor proteins or their ATPase domains have been obtained for bacteriophages T4, Sf6, P74-26, D6E, phi29, and herpes simplex virus 1, as well as evidence that the motor ATPases form a multi-subunit ring surrounding the DNA, and have led to the development of several models for various aspects of motor function (24–29). High-resolution optical tweezers measurements of the phage phi29 motor revealed that DNA is translocated in bursts of four 2.5 bp steps and general features of the motor’s chemo-mechanical kinetic cycle have been described (7,30–31). In several systems, coordination of the multiple motor subunits has been shown to be regulated by trans-acting ‘arginine finger’ residues (26–28). Studies on the effects of site-directed residue changes in the phage T4 and lambda motors have illustrated the roles of specific amino acids in ATP binding, catalysis of hydrolysis, motor force generation, motor pausing and slipping and chemo-mechanical coupling (32–37).

However, fundamental questions about motor–DNA substrate interactions and how they influence various aspects of the packaging mechanism remained poorly understood. First, is the function of packaging motor influenced by DNA sequence in terms of translocation velocity and motor dynamics such as pausing and slipping? Second, do specific DNA properties such as propensity for transition from B- to A-form or local curvature affect the function? In the present work, we use the T4 motor to investigate these questions. Additional motivation for addressing these questions came from several previous experimental findings and one theoretical model that implicate potential sequence effects in motor–DNA interactions during DNA translocation.

First, a striking feature of the T4 motor is that the rate of DNA translocation, even at low capsid filling where forces resisting DNA confinement are negligible, was found to be highly variable (5). The speed of individual motors was observed to vary in time, often by at least several hundreds of bp/s. The cause of these fluctuations is unknown, but since segments with varying DNA sequences were being packaged one hypothesis is that the local sequence of the substrate DNA affects translocation speed. DNA sequence is known to affect local structure and physical properties of DNA including intrinsic curvature, bendability, duplex stability, etc. (38–40). An example relevant to our present studies is that DNA sequence has been shown to affect propensity of DNA to transition between B- and A-forms (41,42), and this propensity is an important factor in some protein–DNA interactions (43–45). Additionally, some aspects of motor function have also been shown to depend on the substrate DNA sequence. For instance, the phage lambda motor is able to recognize a termination sequence in the DNA, which causes the motor to stop translocating and switch to an endonuclease mode (46). Other examples include the bacterial dsDNA translocating chromosome segregation motors FtsK and SpoIIIe, which can change translocation direction in response to certain sequences (47,48).

A second motivation for investigating substrate sequence dependence is the unexplained observation that all three viral motors for which translocation dynamics have been measured exhibit abrupt pauses and slips, even at low capsid filling (4–6). Since these studies measured packaging of heterogeneous DNA sequences, one possibility is that pauses or slips occur, or occur with increased probability, when the motor interacts with certain DNA sequences. An alternate possibility is that these events are independent of DNA sequence and just caused by fluctuations in the conformation of the motor protein and/or its alignment with the DNA that are stochastic in time.

A third motivation for these studies is evidence from single-molecule studies that the phage phi29 motor makes contacts with the DNA bases or sugars during translocation (49). Responses of motor translocation to a range of modified DNA substrates, including ones that contained uncharged methylphosphonate DNA strands, segments with sugars and bases removed, single-stranded gaps or unpaired bulges, were studied as a function of applied load forces. During the dwell phase when ATP binds to multiple motor subunits, important gripping contacts are made with the phosphate backbone, but during the subsequent burst of translocation steps, nonspecific contacts are made with many parts of the DNA including the bases or sugars (49). It is therefore plausible that different DNA base sequences could cause differences in the translocation dynamics.

**Figure 1.**
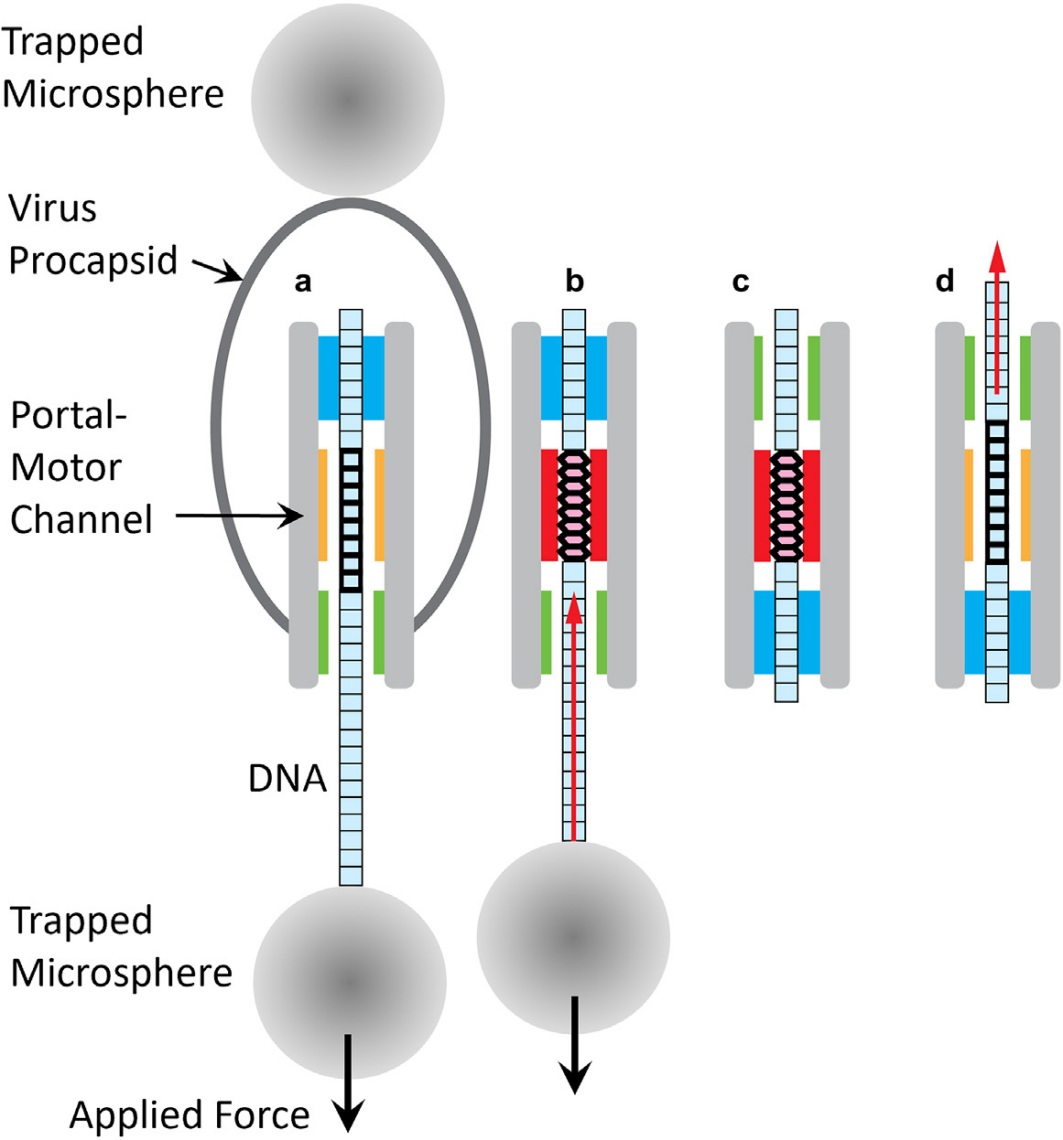
Schematic illustration of the single DNA molecule packaging measurement and B-A scrunchworm model (modified from S. Harvey, *J. Struct. Biol*.**189** (2015); note that the elements are not drawn to scale). (**A**) The viral procapsid-motor complex is attached to one trapped microsphere and the unpackaged end of the DNA is attached to a second trapped microsphere. Translocation by the motor pulls the microspheres together and a resisting force is applied as indicated. The hypothetical mechanism proposed by the B-A scrunchworm model is also illustrated. (**B**) The portal/motor induces a threaded section of DNA to transition to a shortened A-form structure while an upper section is gripped, which results in ∼2.5 bp of DNA being pulled into the channel. (**C**) The upper section of DNA is released, and a lower section of DNA is gripped. (**D**) The grip is released, and the threaded DNA is induced to transition to the longer B-form structure, resulting in translocation of ∼2.5 bp of DNA into the procapsid. The portal/motor then cycles back to state (A). This model may be contrasted with other types of models in which the DNA is assumed to have a static structure and conformational changes in the motor protein complex are proposed to drive DNA translocation steps.

A fourth motivation is that one proposed model for motor function, the ‘B-A scrunchworm’ model hypothesized that the motor induces structural changes in the DNA substrate during translocation which would be sensitive to DNA sequence (50). This model hypothesized that the motor generates force that drives translocation by inducing repeated transitions between the B-form and A-form of DNA segments threaded into the motor channel, coordinated with motor gripping and releasing actions (Figure 1). Transition of the threaded segment from the longer B-form to a shorter A-form was hypothesized to pull ∼2.5 bp of DNA outside the capsid into the motor channel and transition of the segment back to B-form is then proposed to push DNA into the capsid. This model is consistent with experiments that find evidence for DNA length changes within the motor (51,52). It is also in agreement with the experimentally measured phi29 motor step size (7,30–31). This model further argues that the force generated to pull the DNA into the motor channel should depend on the ease with which particular segments of the DNA can convert between the B- and A-forms, characterized by the free energy difference per basepair (Δ*G*_BA_) between B and A forms, which depends on DNA sequence (41). A prediction of this model is that ‘A-philic’ sequences that convert more easily to A-form (having lower Δ*G*_BA_ values) would be easier for the motor to pull in and less affected by external forces opposing translocation. Because motor velocity depends on load force, changes in Δ*G*_BA_ would be expected to alter DNA translocation rate if the B-A scrunchworm model is correct (6,50).

Features of the B-A transition have been reviewed previously (53,54) and we mention only a few. In solution, high concentrations of ethanol can induce B-A transitions (55) and dynamic measurements suggest that the transition timescale is ∼2 μs, although evidence was found for minor relaxation components as long as 50–100 μs (56). This timescale is compatible with the B-A scrunchworm model, which predicts a 2.5 bp step, because it would allow for translocation speeds up to ∼2.5 bp/100 μs = 25 000 bp/s, which is higher than the measured rate. Some studies have also found evidence for intermediates between the A- and B-form DNA that more detailed scrunchworm models may need to consider (57). One puzzling finding is that a study of single DNA stretching in ethanol unexpectedly found no detectible transitions between the ∼0.34 nm/bp length expected for normal B-form DNA and the shorter length (∼0.26 nm/bp) conventionally expected for A-form DNA (58). This study raises questions about the orthodox view that A-form DNA in solution has an inherently shorter length per bp than B-form. However, the B-A scrunchworm model proposes that it is interaction of the portal and/or motor protein rings with the threaded DNA segment that induces a transition to a shorter A-form DNA conformation. The plausibility of such a mechanism is supported by published structures of protein–DNA complexes that show that protein–DNA interactions can induce a shortened A-form DNA conformation (43,59).

As mentioned above, ‘internal forces’ resisting DNA confinement also resist translocation of DNA segments into the viral prohead and exert load force on the motor. The B-A scrunchworm model predicts that it would be more difficult for the motor to translocate A-philic segments into the capsid against internal forces because they make transition of the threaded segments from A-form to B-form more difficult. However, in the early stages of packaging that we study here, where capsid filling is low, internal forces resisting translocation are very small and are expected to have a negligible effect on translocation dynamics (6,14–15,30). Our present studies focus on predicted effects of an externally applied load force (Figure 1), where A-philic segments are predicted to be translocated at a higher rate. Motor velocity slows with increasing applied force (6) and this implies that the rate limiting step in the motor’s mechanochemical cycle is the translocation step (60–62). Translocation rate is predicted to follow Arrhenius-like kinetics and depend exponentially on the height of the energy barrier between the two states (60–62). When the motor generates force to translocate against a load force, this is predicted to raise the energy barrier by an amount *F*Δ*x* and slow the translocation rate by a factor exp(–*F*Δ*x*/*kT*). Since the B-A scrunchworm model proposes that motor force is proportional to Δ*G*_BA_, a reduction in Δ*G*_BA_ is predicted to increase motor velocity.

Here we ask, by placing an A-philic, GC-rich, DNA sequence in the middle of a non-A-philic linear plasmid DNA sequence, whether viral packaging motor velocity and translocation dynamics such as pausing and slipping are altered due to structural differences caused by these sequences. In addition, we ask more generally whether any measured aspects of motor translocation dynamics are affected by DNA sequence. We present measurements of the packaging of different phage and plasmid DNA sequences as well as correlation analyses to look for sequence dependences of packaging dynamics when repeatedly packaging the same sequence versus when packaging different sequences. Our studies show no evidence that either A-philic or other variable sequences influence motor function, aside from small differences in slipping. Our findings therefore suggest that viral genome packaging is insensitive to DNA sequence, impose constraints on the packaging models for motor function, and provide evidence that motor velocity fluctuations, pausing and slipping are primarily stochastic in time.

## MATERIALS AND METHODS

### DNA constructs

A 2014 bp ‘A-philic’ dsDNA sequence, derived from a 40 bp ‘LilF’ sequence described previously (63), was synthesized and cloned into a 9276 bp plasmid vector pPIC9K using NotI and EcoRI cloning sites (ThermoFisher Scientific, Inc., Project ID: 15ADFYGC, Construct Name: t1_lowF-seq). The construct was verified by sequencing and the sequence is provided in the Supplementary Figure S1.

A linear 11270 bp dsDNA construct used as a substrate for packaging was prepared by PCR from this plasmid using the following primers: biotin-5′-ATGAGTGACGACTGAATCCGGTGA-3′ (forward) (IDT, Inc.) and digoxygenin-5′-GGTTGTATTGATGTTGGACGAGTCGGAA-3′ (reverse) (Eurofins Genomics, Inc.) using the LA Taq PCR kit (TaKaRa, Inc.). The biotin label is used to tether the DNA to streptavidin coated microspheres. The digoxygenin label was used for control experiments in which the DNA alone, in the absence of the prohead–motor complex, is tethered to anti-digoxygenin coated microspheres as described previously (64).

The 20049 bp dsDNA ‘control’ non-A-philic construct with 51.8% GC content, used in control experiments, was prepared by PCR from lambda phage DNA (NEB, Inc.) using primers Biotin-5′-CTGATGAGTTCGTGTCCGTACAACTGGCGTAATC-3′ (forward) (IDT, Inc.) and Digoxygenin-5′-GTGCACCATGCAACATGAATAACAGTGGGTTATC-3′ (reverse) (Eurofins Genomics, Inc.) with the LA Taq PCR Kit (TaKaRa, Inc.).

### Packaging measurements

T4 phage capsids and the gp17 motor protein were prepared, complexed and tethered to microspheres as described previously and optical tweezers measurements were conducted using the methods described previously (6,35,65–66). All the measurements were done in a solution containing 50 mM Tris-HCl pH 7.5, 5 mM MgCl_2_, 80 mM NaCl, 0.05 g l^−1^ BSA and 1 mM ATP. The optical tweezers instrument was configured and calibrated as described previously (67,68). Due to variation in the sizes of individual microspheres, there is uncertainty of ±135 nm (about ±400 bp) in the absolute length of DNA packaged (95% confidence interval). All measurements were recorded in ‘force-clamp’ mode (66), in which a feedback control system operating at 1 kHz adjusts the separation between the optical traps in 0.5 nm increments to keep the measured force constant. In the shown DNA length packaged versus time plots, these data were smoothed with a 50-point moving average to reduce noise.

### Motor velocity and pausing/slipping analyses

DNA packaging rate versus time was calculated, as in previous studies (65), by linear fitting of the length versus time data in a 0.5 s sliding time window slid in 5 ms increments. Negative control data in which fixed length DNA molecules, tethered without the head–motor complex, were recorded to determine the effect of noise/drift on the measurements that were analyzed in the same manner. The term ‘motor velocity’ is used to refer to DNA translocation rate during active translocation, omitting periods where pauses or slips occurred. As in previous studies, sections of data in which active translocation occurred were identified based on a velocity threshold criterion considering the effect of noise/drift measured in control experiments with fixed-length tethered DNA molecules (65). Windows in which rate was >55 bp/s were scored as active translocation (97.8% confidence) and sections with rate <55 bp/s were scored as slipping (97.8% confidence). Average velocities (for packaging specified DNA segments with specified applied forces) were calculated by averaging velocities in all time windows scored as having active translocation over all events. Uncertainties in average motor velocity were estimated using the bootstrap method (69).

### Packaging rate fluctuation analyses

Fourier transforms of DNA length packaged versus time were calculated for each event using the FFT function in Matlab (R2019, Mathworks, Inc.). The average FFT was calculated by averaging computed FFT amplitudes in frequency bins over all events. The same analysis was done for the negative control datasets (measurements on fixed length DNA tethers) to characterize the measurement noise. Signal-to-noise ratio (SNR) was calculated by dividing the average FFT amplitudes calculated for signal to those calculated for the control datasets.

### Correlation analyses for packaging rate

Packaging rate versus position along DNA template was calculated by linear fitting of the DNA length versus time data in a sliding 500 bp window, slid in steps of 5 bp. Correlations between rates measured in one event versus another event were analyzed by calculating Pearson correlation coefficients. The following procedure was implemented to account for the effect that the roughly ±400 bp uncertainty in absolute position would have on the detection of correlations. The correlation coefficient for each pair of events was recalculated after shifting one of the datasets by ±100, ±200, ±300 and ±400 bp and the maximum value was determined for each pair. If two events are statistically correlated this shifting procedure would detect the larger correlation that would occur when pairs are more closely aligned in absolute DNA position. These maximum correlation coefficients were determined for all pairs of datasets and averaged to define the ‘correlation score’. By definition, Pearson correlation coefficients have values between -1 and 1 and would average to zero for a large ensemble of pairs of completely uncorrelated datasets. Because we calculate the maximum correlation coefficients for different position shifts, we obtain non-zero positive average values for the correlation scores. To determine whether these values indicate statistically significant correlations we compare correlation scores when correlating pairs of events recorded with the same sequence versus when cross-correlating pairs of events recorded with different sequences. Datasets recorded for the linear ∼11 kbp plasmid sequence and the ∼20 kbp phage sequence, both linear molecules, were used. When cross-correlating, data recorded with the first ∼10 kbp of the plasmid sequence were correlated against data recorded with either the first 10 kbp or second 10 kbp of the phage sequence.

### Correlation analyses for pausing and slipping

Analyses for correlations between pauses and slips occurring in pairs of different events were conducted using a similar approach as that described above used to analyze correlations in packaging rate. Specifically, we first computed pausing and slipping frequencies versus position in a sliding 500 bp window for each event. Then correlation scores were computed for pairs of these records in the same manner as described above.

## RESULTS

### Single molecule design to analyze sequence dependence of packaging motor function

We used optical tweezers to measure motor-driven packaging of single DNA molecules into single phage T4 heads using the techniques we developed previously (6,35,66). In brief, a head–motor complex is attached to one microsphere held in one steerable optical trap and a DNA molecule is attached by one end to a second microsphere held in a second optical trap. To initiate packaging, the two microspheres are brought into near contact in a buffer solution containing ATP, to allow the motor to grip the free end of the DNA and begin translocating it into the head. To test for translocation, we move the two microspheres apart while measuring the force on the second microsphere. If a DNA is tethered, the force rises as it is pulled taut between the two microspheres. We then turn on a ‘force-clamp’ feedback control system which adjusts the separation between the two traps to maintain a specified constant applied force. If the motor is translocating the DNA, a rapid decrease in the separation between the two traps is observed as the two microspheres are pulled closer together. In this manner, we track the length of the DNA packaged versus time under a constant applied load force (external force opposing translocation).

**Figure 2.**
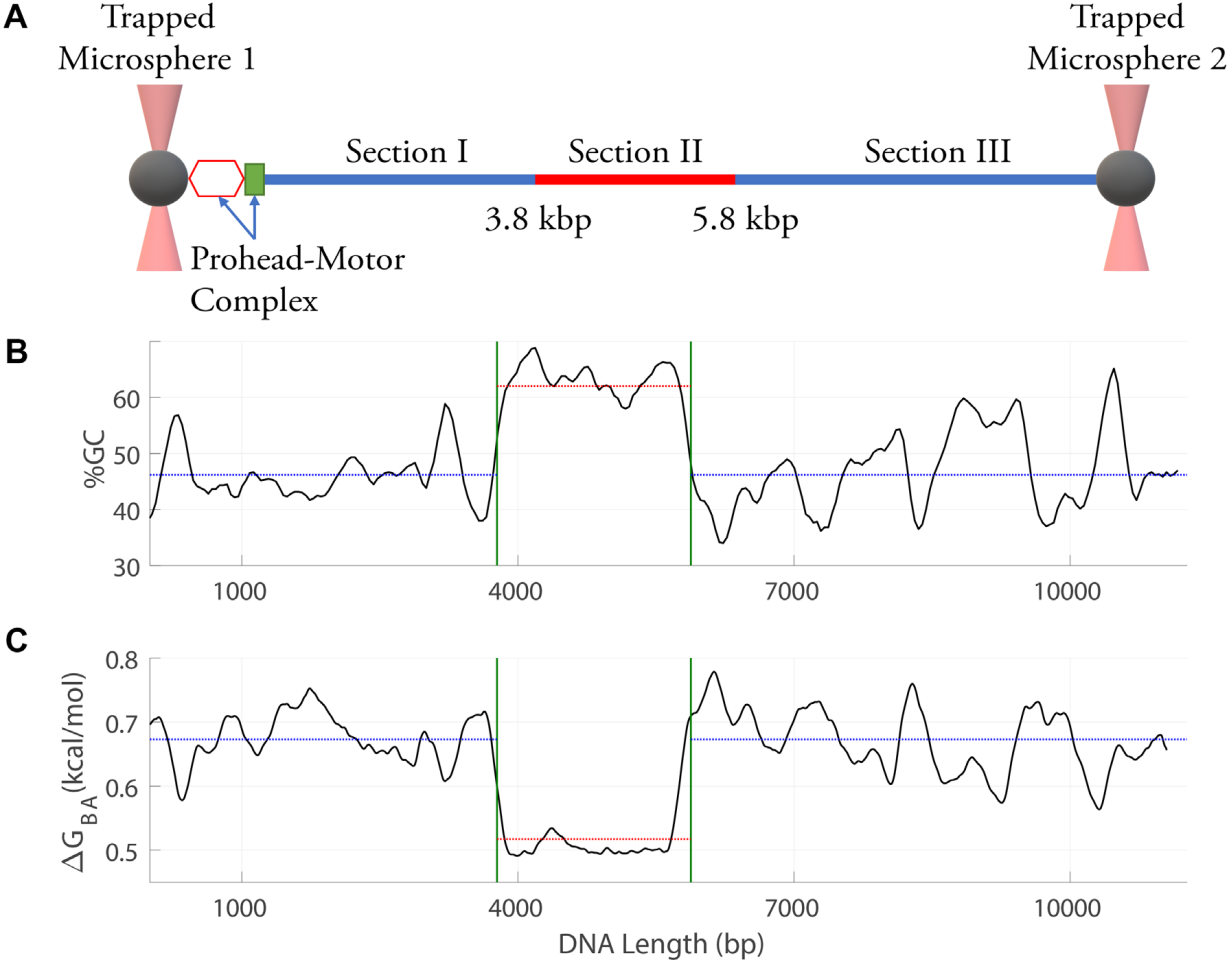
Schematic illustration of the DNA construct design and packaging measurement. (**A**) T4 prohead–motor complexes are attached to one microsphere trapped with optical tweezers (left) and DNA is attached to a second trapped microsphere (right). A linear plasmid DNA sequence was designed with a synthetic A-philic, high GC content sequence (section II) between two ‘normal’ non-A-philic flanking plasmid DNA sequences (sections I and III). DNA translocation by the motor begins at left and proceeds from section I to II to III. (**B**) Average % GC versus position along the DNA calculated in a 200 bp sliding window. (**C**) Predicted free energy difference per basepair (Δ*G*_BA_) between B-form and A-form DNA structures versus position calculated in a 200 bp sliding window. In panels (B) and (C), the vertical lines indicate the beginning and end of section II, the horizontal lines indicate average %GC and Δ*G*_BA_ for sections I and III, and the dashed red line indicates average %GC and Δ*G*_BA_ in section II.

To test whether DNA sequence would affect motor function, and to test the B-A scrunchworm model specifically, we designed a linear DNA construct containing a ∼2 kbp synthetic A-philic sequence inserted into the middle of a ∼9 kbp ‘normal’ (non-A-philic) plasmid DNA sequence, illustrated schematically in Figure 2A. This approach allowed us to measure the effects of sequence within a single packaging event, especially when the motor transitions from the flanking plasmid segments into the A-philic segment, or vice-versa.

Our synthetic sequence was designed to be A-philic based on principles determined by experiments by Minchenkova *et al.* (55), Ivanov *et al.* (70) and Tolstorukov *et al.* (41) which measured B-A transitions for 24 different 9–14 bp sequences to determine how Δ*G*_BA_ depends on sequence. These studies established that both high GC content and the presence of certain dimers and trimers cause sequences to be more A-philic. The results were shown to be well fit by an empirical ‘T-32’ model with experimentally determined parameters (41). The B-A scrunchworm model, which predicts that a ∼10 bp segment in the motor channel is induced to transition to A-form, specifically proposed that these Δ*G*_BA_ values for short DNA segments are relevant to motor function (50). The A-philic sequence we designed has ∼64% GC content whereas the flanking plasmid DNA sequence has only ∼46% (Figure 2B). In addition, 60% of the included dimers are ones classified by Tolstorukov *et al.* as A-philic (versus 53% of those in the flanking plasmid DNA) and 33% of the included trimers are ones classified as highly A-philic (versus only 14% in the flanking plasmid DNA). The Δ*G*_BA_ values calculated with the T-32 model for this sequence are significantly lower than for the flanking plasmid DNA sequences (Figure 2C), implying that transition to A-form is more easily induced. In the B-A scrunchworm model, translocation of A-philic DNA segments is predicted to be less-inhibited by an applied load force.

### A-philic DNA sequences do not alter motor velocity

**Figure 3.**
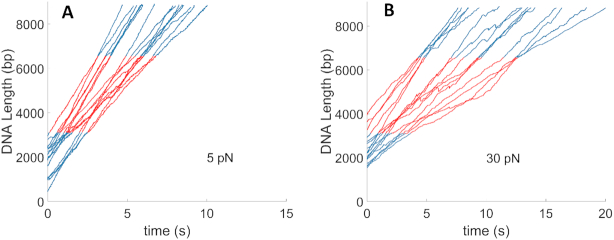
Measurements of length of DNA translocated versus time with the linear plasmid DNA sequence. Parts of the dataset containing the non-A-philic flanking plasmid sequences (sections I and III) are indicated in blue (see online figure) and parts containing the synthetic A-philic, high % GC sequence (section II) are indicated in red (see online figure). (**A**) Measurements with 5 pN applied force (examples from *N* = 134 recorded events) (**B**) Measurements with 30 pN applied force (examples from *N* = 45 recorded events).

Single DNA molecule packaging measurements with the linear plasmid DNA sequence were made at saturating ATP concentration (1 mM) using both low applied force (5 pN), where the motor translocates DNA at nearly maximum speed, and high applied force (30 pN), where the motor is slowed by ∼60% (6). About 30 pN is the estimated maximum internal force the motor experiences during the end stages of packaging the full-length viral genome (14,30,71–72). We collected *N* = 134 packaging events at 5 pN and *N* = 45 events at 30 pN. It is considerably more difficult to obtain measurements with the higher force because tethered complexes often detach from the microspheres before the measurement is completed, likely due to dissociation of the antibody-capsid bonds (64). Examples of measured length of DNA packaged versus time records are shown in Figure 3A and B. From these datasets one can see that there is variability in the motor velocity, as we have reported previously. However, there are no obvious changes when the motor transitions from the flanking plasmid section (blue) into A-philic (red) section, or vice versa.

**Figure 4.**
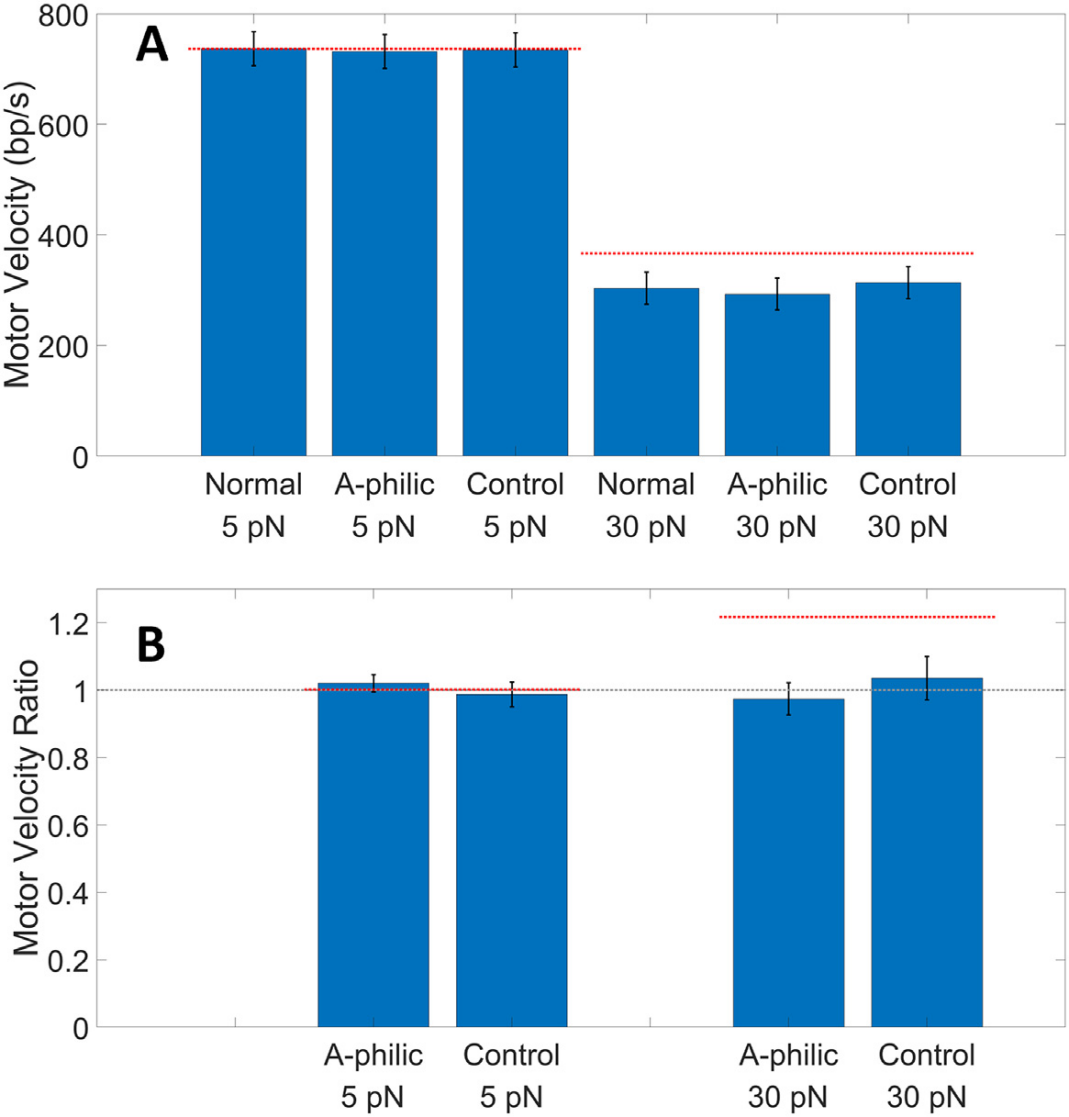
(**A**) Average motor velocities. ‘Normal’ refers to measurements with the non-A-philic flanking sections I and III of the linear plasmid DNA sequence, ‘A-philic’ to section II, and ‘Control’ to the phage DNA sequence. Averages were determined from 134 recorded events at 5 pN and 45 recorded events at 30 pN for the linear plasmid sequence and 80 events at 5 pN and 50 events at 30 pN for the control DNA. Error bars indicate standard errors in the means. The horizontal lines indicate the velocity for the A-philic sequence predicted by the B-A scrunchworm model and force–velocity relationship. (**B**) Average velocity ratios. Bars labeled ‘A-philic’ refer to the average ratio of the velocity when packaging the A-philic segment to that when packaging the normal flanking plasmid segments calculated for each event. Bars labeled ‘control’ refer to velocity ratios calculated in the same manner for events recorded with the phage DNA. Error bars indicate standard errors. The dashed gray line indicates a ratio of ‘1’, expected if there is no sequence dependence. The horizontal lines indicate ratios predicted by the B-A scrunchworm model and measured force–velocity relationship.

We analyzed the full ensemble of packaging events and found no significant differences in the average motor velocity when packaging the flanking plasmid versus A-philic segments at both low (5 pN) and high (30 pN) applied forces (Figure 4A and Table 1). As in our previous work (6), we define ‘motor velocity’ as the rate of DNA translocation during active packaging, i.e. excluding pauses and slips. Additional ‘negative control’ measurements were conducted using a ∼20 kbp phage DNA construct (Supplementary Figure S2). Like the non-A-philic sections of the flanking plasmid sequences, this sequence has much lower average GC content (51.8%) and a much higher average Δ*G*_BA_ (0.6487 kcal/mol) than the synthetic A-philic sequence. We again found no significant differences in average motor velocity (Figure 4A and Table 1).

**Table 1.** Measured parameters characterizing motor function. Each parameter is an average over all events and uncertainties are reported as standard errors. ‘Normal’ refers to measurements with the non-A-philic flanking sections I and III of the linear plasmid DNA sequence, ‘A-philic’ to section II and ‘Control’ to the phage DNA sequence

DNA Segment and applied force	Average velocity (bp/sec)	Velocity ratio	% Time pausing	% Time slipping	# of events
**A-philic, 5 pN**	731.85 ± 30.51	1.02 ± 0.02564	1.63% ± 0.57%	2.36% ± 0.59%	134
**Normal, 5 pN**	736.9 ± 30.88	1 (by definition)	2.88% ± 0.66%	1.02% ± 0.46%	134
**Control, 5 pN**	734.6 ± 30.7	0.9870 ± 0.0364	1.60% ± 0.17%	2.97% ± 0.2%	139
**A-philic, 30 pN**	293.09 ± 28.85	0.9739 ± 0.0478	13.10% ±1.41%	9.38% ± 1.14%	45
**Normal, 30 pN**	303.35 ± 29.17	1 (by definition)	12.29% ± 1.18%	11.43% ± 1.11%	45
**Control, 30 pN**	313.7 ± 29	1.0349 ± 0.0644	11.59% ± 1.32%	15.85% ± 2.3%	51

Uncertainties in the determination of the average velocities were ∼4% for the 5 pN measurements and ∼10% for the 30 pN measurements. These uncertainties are mostly due to the fact that, as we reported previously, different individual T4 packaging events exhibit different average motor velocities (6). Thus, an alternative and more sensitive test is to examine the ratios of motor velocities measured during each packaging event when a single motor packages through A-philic versus flanking plasmid sections of the same DNA molecule. We calculated the ratios of motor velocity when packaging the A-philic segment to that when packaging the flanking plasmid segments for each event and then calculated the average ratio over all events. For both the 5 and 30 pN data, we find that the average ratio is close to unity (Figure 4B and Table 1), again providing evidence that there is no significant effect of the A-philic sequence on motor velocity. As expected, the uncertainties in the ratios are much lower, ∼2.5% for the 5 pN data and ∼5% for the 30 pN data, and thus establish stricter bounds on the null effect of sequence. As a control, we also calculated ratios in the same manner for motor velocities measured when packaging segments of the control phage DNA (which has no A-philic section) starting/ending at the same positions that delineate the flanking plasmid versus A-philic sections in the DNA sequence. As expected, the average ratios determined by this analysis are close to unity (Figure 4B and Table 1).

### Effects of A-philic DNA sequences on motor pausing and slipping

As reported in previous studies, T4 and other phage packaging motors exhibit occasional pauses where the translocation stops transiently, and slips, where the motor transiently loses grip on the DNA resulting in rapid release of packaged DNA under the applied force (6,65). To investigate whether the substantially different A-philic sequence affects pausing or slipping, we analyzed the data to determine the percent time the motor paused or slipped during packaging at both 5 and 30 pN applied forces (Table 1). No significant differences were observed in pausing between the A-philic, flanking plasmid and control sequences with either applied force. However, modest differences were detected in percent time slipping (Table 1). The largest difference in percent time slipping was 9.38% ± 1.14% for the A-philic DNA versus 15.85% ± 2.3% with the control phage DNA sequence with the higher 30 pN force. Although modest, this difference is statistically significant (*P* = 0.007) suggesting that the strength of the motor’s grip on DNA can be affected by DNA sequence. However, it is important to note that this difference does not cause a significant change in the overall rate of DNA translocation.

### Tests for general sequence dependence of motor function

**Figure 5.**
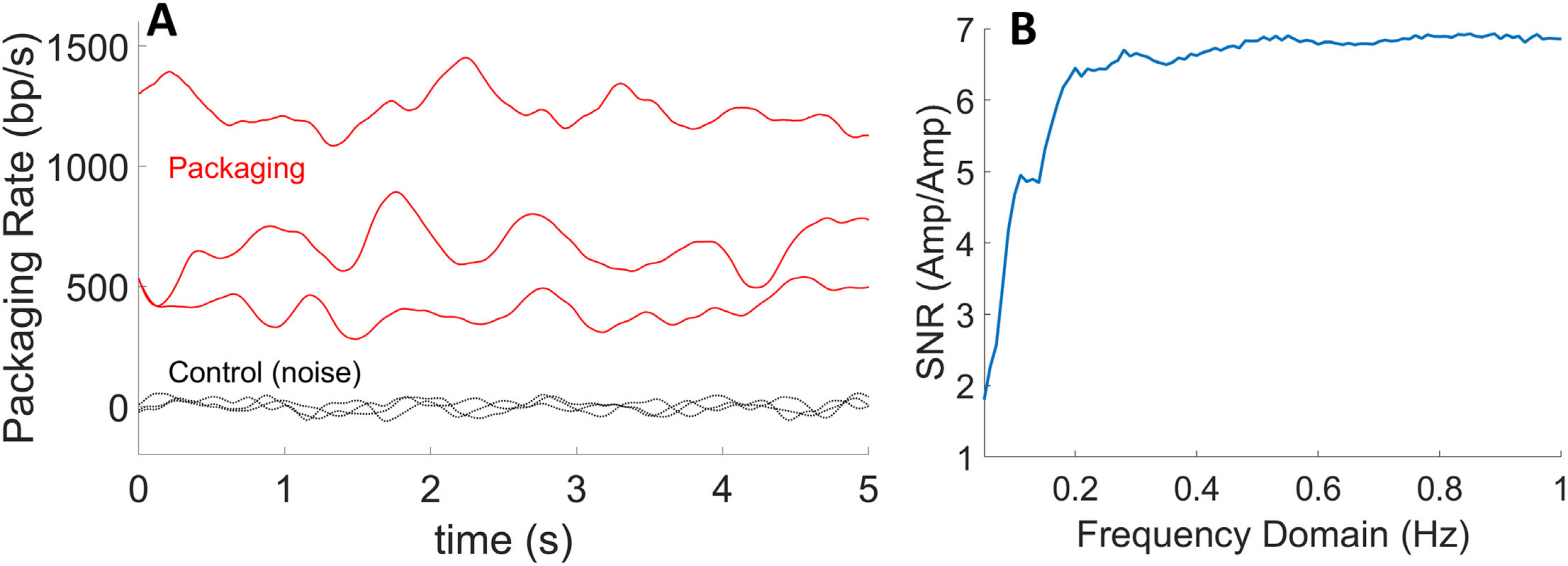
(**A**) Examples of measured packaging rate versus time with the linear plasmid DNA sequence with 5 pN applied force (top three lines). Shown for comparison are rate fluctuations caused by Brownian and instrumental noise measured in control experiments with fixed tethered DNA molecules (bottom three lines). (**B**) Signal-to-noise ratio versus frequency determined by calculating the average Fourier transform amplitudes for packaging rate measurements (‘signal’) (from *N* = 134 packaging events) and dividing them by the average amplitudes calculated from control (‘noise’) rate measurements.

The data presented above provide evidence that an A-philic DNA sequence with high GC-content does not significantly affect motor function. We also sought to investigate if any DNA sequence differences, aside from A-philic propensity or GC content, could influence motor function. As reported previously, the T4 packaging rate fluctuates considerably not only between different packaging events but also during each packaging event (6). Examples of the latter are shown in Figure 5A. To demonstrate that these fluctuations are not simply due to measurement noise/drift, we conducted control force-clamp measurements with fixed-length DNA molecules tethered between microspheres (in the absence of head–motor complexes and thus the absence of DNA translocation) to characterize the instrumental noise/drift. These measurements show that fluctuations in measured translocation rate caused by noise/drift are much smaller than those observed during DNA packaging (Figure 5A).

**Figure 6.**
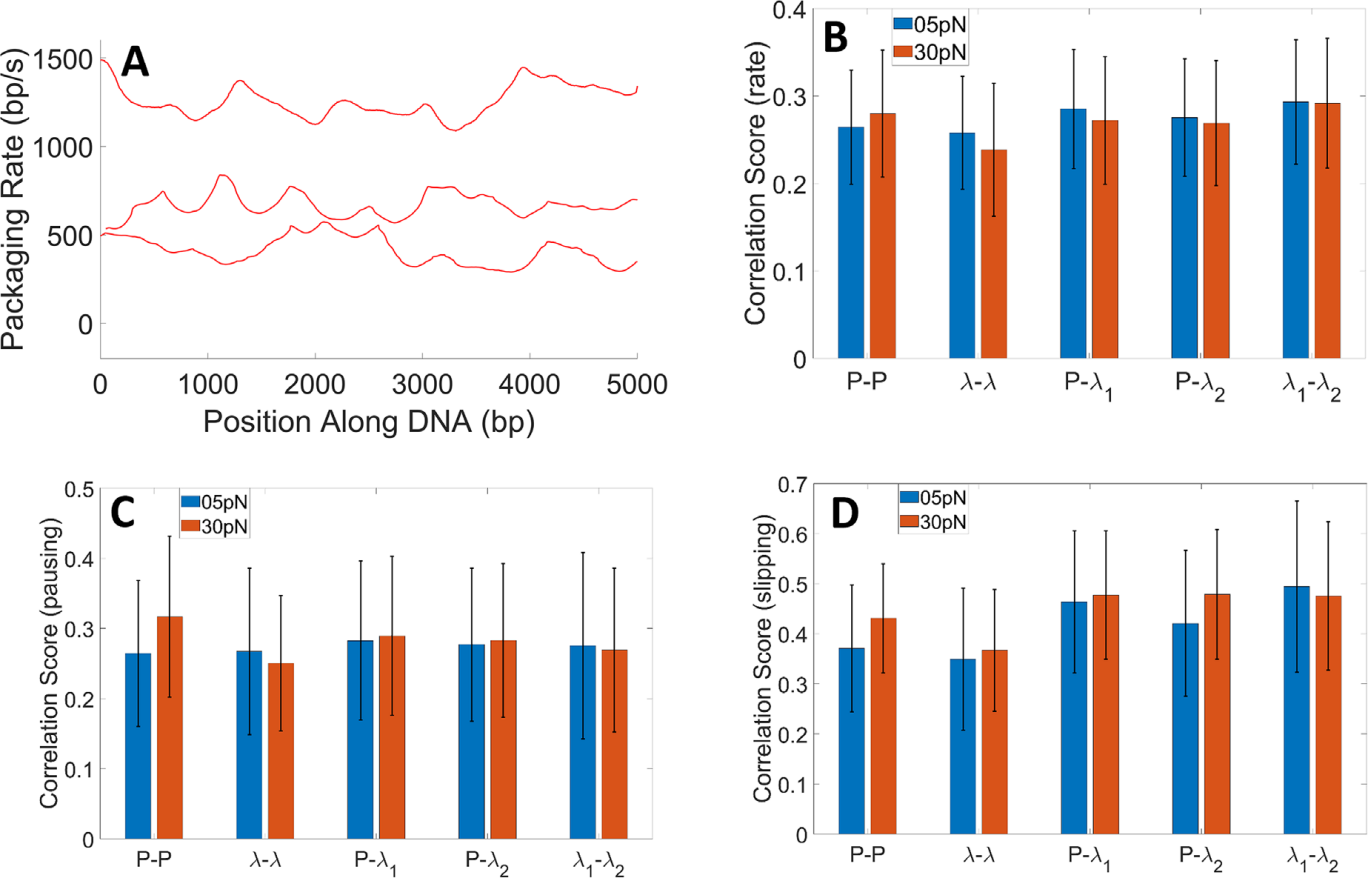
Correlation analyses. (**A**) Examples of measured packaging rate versus position along DNA measured with the linear plasmid DNA sequence. (**B**) Correlations analyzed between rate versus position along DNA measured in one event and that measured in other events when packaging either the same sequence or different sequences. ‘Correlation score’ is defined in the methods section. ‘P’ refers to the linear plasmid DNA sequence, λ to the control phage DNA, λ_1_ to one 10 kbp section of the control DNA, and λ_2_ to another 10 kbp section of the control DNA (see ‘Materials and Methods’ section). ‘P-P’ indicates correlation score for pairs of events where P was packaged. ‘λ-λ’ refers pairs of events where λ was packaged. ‘P-λ_1_’ and ‘P-λ_2_’ refer to correlations between P datasets and λ_1_ or λ_2_ datasets. ‘λ_1_-λ_2_’ refers to correlations between λ_1_ and λ_2_ datasets. Results were determined from 134 recorded events at 5 pN and 45 recorded events at 30 pN for the linear plasmid sequence and 80 events at 5 pN and 50 events at 30 pN for the control DNA. Error bars for all plots indicate one standard deviation. (**C**) Correlations analyzed between pausing versus position measured in one event and that measured in other events, as in (B). Correlation score for pausing is defined in methods. (**D**) Correlations analyzed between slipping versus position measured in one event and that measured in other events, as in (B). Correlation score for slipping is defined in ‘Materials and Methods’ section.

To characterize the timescales of the translocation rate fluctuations, we computed Fourier transforms (FFTs) averaged over all datasets, and then signal-to-noise ratio (SNR) versus frequency by dividing the average FFT amplitudes for the packaging data by the average FFT amplitudes calculated for the control noise/drift measurements (Figure 5B). This analysis shows that the inherent packaging rate fluctuations span frequencies from at least ∼0.05 to 1 Hz. The cause of these fluctuations is unknown, but one hypothesis is that they could be caused by variations in the substrate DNA sequence. To test this hypothesis, we first calculated packaging rate versus position along DNA for each packaging event (Figure 6A) and then looked for correlations in the fluctuations across different events when packaging the same sequence versus different sequences. We used datasets of ∼10 kbp recorded with the linear plasmid DNA sequence and two different segments of the longer phage DNA.

An important consideration in this analysis is accounting for the effect of uncertainty in the measurement of absolute lengths of DNA packaged, which is about ±135 nm or ±400 bp. This uncertainty is caused by variations in sizes of individual trapped microspheres. To account for the effect of this uncertainty in the analysis, we defined a ‘correlation score’ that considers the effect of relative position shifts between pairs of data sets (see ‘Materials and Methods’ section). An important control in this analysis is comparing correlation scores across pairs of events when packaging the same sequence with those across pairs of events when packaging different sequences. Together, these analyses covered a large variety of sequence space since three different ∼10 kbp-long sequences were tested. No significant differences were found in the correlation scores (Figure 6B), and thus no evidence that the packaging rate fluctuations are attributable to DNA sequence. We further investigated the occurrence of pauses or slips using a similar correlation score analysis (see ‘Materials and Methods’ section) and again found no significant differences when correlating pairs of events when packaging the same DNA sequence versus pairs of events when packaging different sequences (Figure 6C and D). Thus, we found no evidence that the pausing or slipping positions are influenced by DNA sequence.

## DISCUSSION

One of the key questions in viral genome packaging is how DNA sequence and/or structure influence packaging motor–DNA substrate interactions and dynamics of translocation. Previous experimental data implicate potential sequence effects, and one theoretical model hypothesized that structural transitions between B- and A-forms might be important for force generation and DNA movement. We addressed these issues through single-molecule optical tweezers measurements of DNA translocation by the phage T4 motor.

Our studies show that there are no differences in average motor velocities of the T4 motor, within the experimental uncertainties, when the motor is packaging A-philic and high GC DNA sequences versus the non-A-philic plasmid and phage sequences. This finding contrasts with the B-A scrunchworm model which predicts that the A-philic sequence would be more rapidly packaged against an externally applied load force. The model predicts that motor force is proportional to Δ*G*_AB_ (50). Since motor velocity decreases with increasing applied force, the predicted effect of the A-philic sequence on motor velocity is calculated by multiplying forces by the ratio of Δ*G*_AB_ calculated for the flanking plasmid sequence to that for the A-philic sequence (6,50). As indicated by the dashed lines in Figure 4, a negligible difference is predicted for the 5 pN force, consistent with our findings, but a significant ∼20% higher velocity is predicted for the A-philic sequence with a 30 pN force. In contrast, our measurements with 30 pN force find an average ratio of velocities for A-philic versus flanking plasmid sections of 0.9739 ± 0.0478, which indicates no significant change is caused by the A-philic, high GC content sequence to within an experimental uncertainty of ∼5%. This finding provides evidence against the B-A scrunchworm model and further shows that GC content does not strongly influence motor function.

Our studies examined translocation dynamics at low prohead filling, where internal forces resisting packaging are negligible. The B-A scrunchworm model predicts that packaging of A-philic DNA would be less-inhibited by an external force but more-inhibited by ‘internal forces’ resisting DNA packaging that occur during the latter stages of capsid filling. We are not presently able to examine this regime because it is technically challenging to manipulate DNA of the full ∼170 kbp T4 genome length with optical tweezers. Based on studies of phage phi29 and theoretical models (14,30,72), internal forces are estimated to rise to ∼20–30 pN near the end of packaging. In this case, the B-A scrunchworm model would predict a lower translocation rate for the A-philic DNA in the late stages of packaging. However, since our present results provide evidence against the B-A scrunchworm model, we do not believe this is a likely outcome. A related consideration is that sequence could influence physical properties such as DNA curvature and bendability and thus potentially influence internal forces by influencing the conformation and dynamics of the packaged DNA (38,73–74). But again, we would expect such effects to only potentially influence motor dynamics in the high capsid filling regime.

After the B-A scrunchworm model was proposed (50), computational studies on DNA within the phi29 connector channel (albeit lacking the packaging ATPase) supported the argument that DNA might be driven to a scrunched conformation (63,75). However, this conformation was even shorter than the standard A-form and the effect was found to be independent of DNA sequence (63), which is consistent with our experimental results. An alternative class of models based on structures of several viral motor proteins proposes that DNA translocation is primarily driven by lever-like conformational changes in the motor protein ATPase (24–26,28). However, it remains possible that a scrunchworm-type mechanism in which expansion and contraction of the threaded DNA segment could play a role in translocation but without involving an A-form DNA structure. Additional and more recent simulations of DNA threaded into the portal rings of phages phi29, T4 and P22 showed that contraction or lengthening of the threaded DNA segment in the channel can occur due to the electrostatic potentials generated by the portal rings (76). This led to a proposed ‘electrostatic scrunchworm’ model. It proposes that ATPase-driven conformational changes in the proteins of the motor/portal complex lead to cyclical changes in the electrostatic potential of the portal channel causing DNA scrunching-unscrunching motions. These are coupled to a protein–DNA grip and release cycle to rectify the DNA motion (76).

Recent structures of the phage P23-45 portal channel (77,78) may have implications for scrunchworm-type models. The channel at its narrowest point was observed to have a different conformation ∼8 Å wider in empty procapsids than in expanded capsids. It was suggested that the portal may have this more ‘open’ conformation during DNA packaging at low procapsid filling and transition to the tighter-fitting conformation at high filling to restrict DNA slipping. It is then possible the portal protein might not interact strongly enough with the DNA to induce structural transitions. However, these studies describe static portal structures in the absence of the motor ATPase and DNA. Interactions between the motor and portal could affect their conformations and interactions with DNA during packaging, or the ATPase could induce DNA transitions instead of the portal.

One recently published study and two preprints provide complementary information consistent with our findings. First, it was shown that 25–35 bp DNA:RNA duplexes can be packaged by the T4 motor (79). Since such duplexes are expected to have A-form structure and not undergo B-to-A transitions, this argues against the B-A scrunchworm model. Second, a recent preprint reports that the phage phi29 motor can also translocate both DNA:RNA duplexes as well as RNA:RNA duplexes and the motor’s step size changes to match their shorter helical pitch (https://www.biorxiv.org/content/10.1101/2020.05.22.101154v1.abstract). Third, a recent preprint reports a cryo-electron microscopy structure of stalled phi29 packaging complex in which the five motor subunits are arranged in a helical ‘lock-washer’ structure with symmetry complementary to the DNA substrate (https://www.biorxiv.org/content/10.1101/2020.05.23.112524v2). It is proposed that the phi29 motor may function by cycling between this helical structure and a planar one, a mechanism that attributes translocation to conformational changes of the motor complex rather than of the DNA. On the other hand, it was noted that the threaded DNA in this structure is ‘stretched or partially unwound in some places, compressed in others, and has a prominent kink’. Whether these features represent dynamic changes in the DNA conformation that could play a role in the motor mechanism remains an open question.

Our measurements revealed modest effects of sequence on motor slipping. A lower percent time slipping was measured with the A-philic, high GC sequence than with the control phage DNA sequence. However, the amount of slipping observed with the flanking sections of the non-A-philic plasmid sequence was similar to that observed with the A-philic sequence, indicating that it is not A-philic property or high GC content that causes the difference. Our findings are consistent with experimental (49) and computational (63) evidence from the phi29 system suggesting that the motor and/or portal proteins make contact with the DNA bases during translocation steps. The detailed nature of these contacts could influence the strength of the motor’s grip on DNA in a manner that depends on the identity of the bases. It has been shown that binding of ATP induces the motor to transition into a conformation where it tightly grips DNA and that slipping can occur due to either transient ATP dissociation or force-induced rupture of the motor’s grip (36,62,65). It is conceivable that either effect could be influenced by the interacting DNA sequence, the former potentially via allosteric effects (80). On the other hand, the small difference in percent time slipping does not result in a significant difference in overall translocation rate. Moreover, slips do not occur at the same positions in every packaging event, and the observed change in percent time slipping with sequence at high force is less than the average percent time slipping. These findings suggest that while slipping propensity can be affected by sequence it is primarily a stochastic temporal process.

No significant differences in motor pausing were observed with the A-philic, GC rich sequence. The cause of motor pausing is not completely clear, but it has been observed with all the three well-characterized motors from T4, lambda and phi29 phages. In the case of T4, pausing was attributed to misaligned DNA in the motor channel when the ATP-binding site was unoccupied (81). Alternatively, studies of lambda motor mutants exhibiting altered pausing suggested that that pausing can occur due to binding of ATP in a misaligned orientation that leads to temporary blockage of hydrolysis (36). In addition, studies of the phi29 motor revealed that an increase in pausing at high prohead filling (>75% of genome length packaged) is attributable to non-equilibrium dynamics of the tightly packed DNA via fluctuating internal forces and/or allosteric regulation of motor function (20,71). This latter effect is not relevant to the present measurements with T4 because we are measuring at low prohead filling. Our finding of no evidence for sequence dependence suggests that pausing is mainly a stochastic temporal process.

Our additional analyses with three different ∼10 kbp DNA sequences found no evidence that packaging rate fluctuations, pausing or slipping were correlated with position along the DNA templates. A limitation of this analysis is that there are limits on the time resolution of measurements of translocation rate because they require calculating the derivative of the DNA length versus time data which are affected by noise. For the analyses in Figure 6, we calculated packaging rate and frequency of slipping and pausing in a sliding 500 bp length window, so these analyses do not address whether shorter sequences could influence packaging dynamics unless such sequences occurred with significantly different probabilities in different 500 bp windows. However, the results in Figure 5B show that large fluctuations in the packaging rate that we sought to explain occur at frequencies from 0.05 to 1 Hz. Since the average DNA translocation rate is ∼700 bp/s, these rate fluctuations occur on DNA length scales ranging from 700 to 14 000 bp, which implies that a 500 bp sliding window is appropriate for the analysis and we can conclude that these fluctuations are not caused by sequence dependence. We also conducted additional correlation analyses using a smaller 200 bp window size and again found no evidence for sequence-dependent packaging dynamics (Supplementary Figures S3 and S4).

Since pauses and slips are abrupt events which likely occur at specific positions along DNA it seems likely they could be influenced by DNA sequences shorter than 200 bp. During translocation the motor likely contacts a small section of the threaded DNA comprising perhaps just ∼1–10 basepairs (49). The probability to slip or pause may vary depending on the identities of those basepairs. Our measurements with the engineered high GC DNA segment provide clear evidence that G-C and C-G basepairs don’t cause major differences versus A-T or T-A basepairs. As mentioned above, differences caused by sequences <200 bp could be detected if the sequences of interest occur with significantly different frequency within different 200–500 bp sections of the DNA template. Any sequences of interest, of any length, could be tested using the methods presented here by using engineered DNA templates containing a section enriched in these sequences.

To investigate whether the synthetic A-philic sequence, we designed exhibits other physical properties distinct from those of the flanking plasmid sequences we used the ‘plot.it’ and ‘bend.it’ software packages (40). Differences in several properties are predicted (Supplementary Figure S5 and Table S1). The most notable is that average roll angle between basepairs (73) is predicted to be ∼5-fold higher for the A-philic sequence. Roll angles can be positive or negative, but for the A-philic segment they are predicted to be predominantly positive. Average twist angle (73) is predicted to be ∼0.6° lower for the A-philic section, which is a significant difference since the standard error in the mean (SEM) is 0.034°. Intrinsic curvature (38) is predicted to be 43% lower (SEM = 6.9%) and average bendability (74) 7% higher (SEM = 0.72%). Average free energy (Δ*G*) of melting (39), a measure of duplex stability, is predicted to be 8% higher (SEM = 1.3%). Since our measurements found no significant differences in DNA translocation dynamics when packaging the A-philic versus flanking plasmid segments, our results provide evidence that these DNA property changes do not significantly affect motor function.

In summary, our analyses of a viral genome packaging motor show that variable DNA sequences do not significantly affect the function of the motor. No influences on motor translocation rate or pausing were detected. Only modest differences in slipping were detected, which suggest that the motor's grip on DNA can vary with sequence, but this does not significantly affect the overall DNA packaging rate. The finding that an engineered A-philic DNA sequence has no significant effect on motor velocity with a high applied load force provides evidence against the B-A scrunchworm model (50). These results do not provide evidence for or against the electrostatic scrunchworm model (76). Overall, our results suggest that motor velocity fluctuations, pausing and slipping are primarily stochastic temporal events. These insights impose constraints on the plausible packaging models for motor function, particularly those requiring structural changes in the DNA substrate for DNA movement. Furthermore, insensitivity to sequence relieves any sequence dependent genome packaging constraints on virus evolution. Otherwise, it could create evolutionary bottlenecks for regulatory sequences that control viral life cycle such as transcription, replication, recombination and repair.

As additional data become available from molecular genetic analyses of the motor proteins, and from high-resolution structural studies of the packaging motor complexes, more detailed models for motor force generation in the phage DNA packaging system will be developed. The results reported here, along with those from other single-molecule experiments (9,31,65,71,81–83), will be critical for evaluating those models, and for developing a complete structural, kinetic and thermodynamic understanding of how these motors work.

## Supplementary Material

gkaa875_Supplemental_File
