# Supplementary material for: Function of a viral genome packaging motor from bacteriophage T4 is insensitive to DNA sequence
Source: Nucleic Acids Res. 2020 Oct 29;48(20):11602–14. doi: 10.1093/nar/gkaa875 (PMC7672480; doi:10.1093/nar/gkaa875)
Supplement: gkaa875_Supplemental_File [file gkaa875_supplemental_file.docx]

**SUPPLEMENTARY DATA**

**Function of a viral genome packaging motor from bacteriophage T4 is insensitive to DNA sequence**

Youbin Mo^1^, Nicholas Keller^1^, Damian delToro^1^, Neeti Ananthaswamy^2^, Stephen C. Harvey^3,*^,

Venigalla B. Rao^2,*^ and Douglas E. Smith^1,*^

^1^ Department of Physics, University of California, San Diego, La Jolla, CA, 92093, USA

^2^ Department of Biology, The Catholic University of America, District of Columbia, USA

^3^ Department of Biochemistry and Biophysics, Univ. of Pennsylvania, Philadelphia, PA 19104, USA

**Fig. S1. Sequence of the synthetic A-philic DNA segment:**

5’‑TATCTCTCGAGAAAAGAGAGGCTGAAGCTTACGTAGAATTCGGCACCTGCGGGCTACCGCGGTACTACCTACCTACACCACCTACACCTATACCCCCCACTACCTACCCCGTACCCCCCCCCTACCGGGGGCCCTACGGCTCCCACACTAGGTGCTACTACACCGTACCACTACCCACCCCCCTACCACGCCCACTACACTCCCTATACCTACCTACTACCCCCACGGTACATCGGTACGGGGGCACCCCCCTATCTGCTACCCCCGCCCCACCTACGCGGGCGGGGGTACCACCCACGGGGTACCCAGCCACACCCCGCTAGCTACCCCTAGCAGGCCACCCCTACCGTAGGTACATACGTACCGCCGCCTACCTACGGCATACCTATACACCCCTACCCCTATGCGCTCCCCCCCCCACACTATACCCCACACCCCCACGATACCGGCCCGCCACTGGGGTACCCCTAGCCACTATAGTACTGGGTACTACCCGCCCGCACCCGGTACCACCCACCCCTATAGTGGAGGTACACTACACACGTCGGCTACGGTACCCACCACGTACTCCCACGGCATACCCACCTACCTATACCGGCCACACACCCTCGGTACCTACCGTACCCCCTATATACCACGGGCATACCTCCCCACCATCCTACCGCACTATACGGCCCCCCCATACATACCTACTACAGTACCCATACCCGGGACCACCACCCCATACACCTGACTCCACCACCCCCGCTACCTATACTACCCGTATACCCTATAGCCCACTACTACTGATATGTACCTACCACCAGCCTACGCCAGGGGGCCCCTGACGTACGCTACCACCCCTACTACCCCCCGGTACGCCCACCCCCCGCCCCGGTATAGGGGGCTATACACCTACTAGTGCCCTACCTACACTACCCACCGGTGGGTACTACCGTATACACCTACTGCCACCTCGGGTATACCACTACCACACCCCCCGTACCTACCCAGACCCCCGGTGTACCCACACCCCTACATACTACTGGCTACGTACCCCTGCTAGTACCCACTACACTACACCCCCACACCGGGTCGCCCTACCGGTGGCCCCCCCACCTGCACCTACCCCACGCCTACTACCCCTAGTAGGCCCTACCCCCTACGTGCATAGCACCCCCCCCCTACCCTACACCCGGGGCCCACCGTGTACCACCCACTAGGGTACCCCCTACGCTACTCCATACTGGCCCCGTATACCCACACCACACACCATACACACCTACTACCGGGTATACGTGGACCACTACTACCACCCCTGCGGTACTAGGCCTCCCCTACCGCCCGCCGCCGGGGGCTACTACCACTACCACCCATACTACCCCACCCTAGGCCTAGGCTACACGTGGACACACCCCCCATCGCCCATACCACGCATGGGCTAGGGCACACCCCCACCCTACCTACTACCCCACTACCCCGGGTACCCCTACTACTACGCACACTAGGCCCCACTACCTGCACACCACACTACCTATACCATAGTAGCGGCCCCACTACGTACTACCACACCCCTACTACCAGCTAGGGCCACCCCTATACCCGGGGGGACATACCGGCCCCCCGGGGGGGGCCGGCCTACCTACCCACTACGGACCACTACCTACACACCTCCCACCCCCCCCACCCTATCCACACCTACCTACGCCGTACGGGGCCTACCGTACCGCCCCCTACCACCTACCCCTACCTCTAGCCCCCAGGGGGGGTACACTAGCCCTCCCCCCATACACCACGTAGGGCCCCCACGTCTGCGGCCCTACATAGTACCTAGGGGCCCCTACCCCCTGGCCCTACCTACTACCCTCGGTACTCACCCCACTATCCCCCCCTACACCCACAGGTACCCTACCCTACCGCTGGCCGGTACGGTACCGCCGCTACTACAGCTGTACTACTACTGGGGGCACCCCCACTATGCACGGGGCTGGTACTACTACGCCTACCCCACTACATACTACCACCCTCCCACGTA-3’


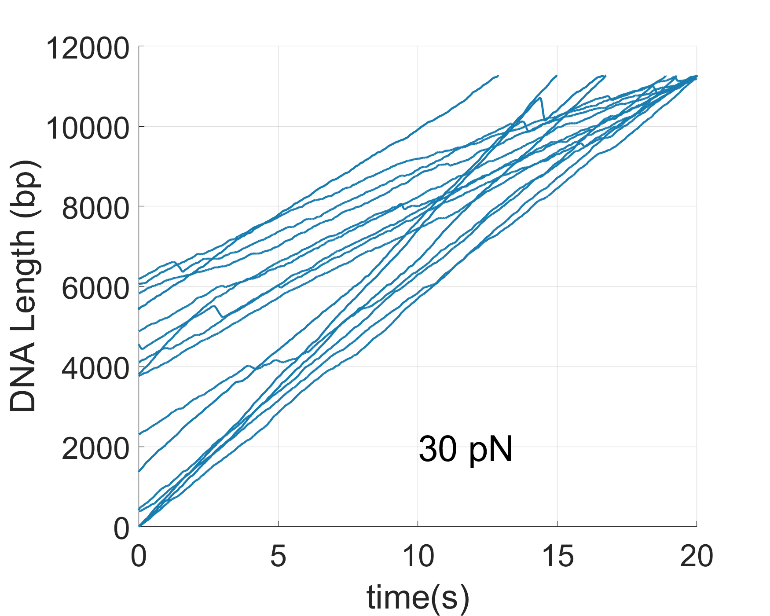


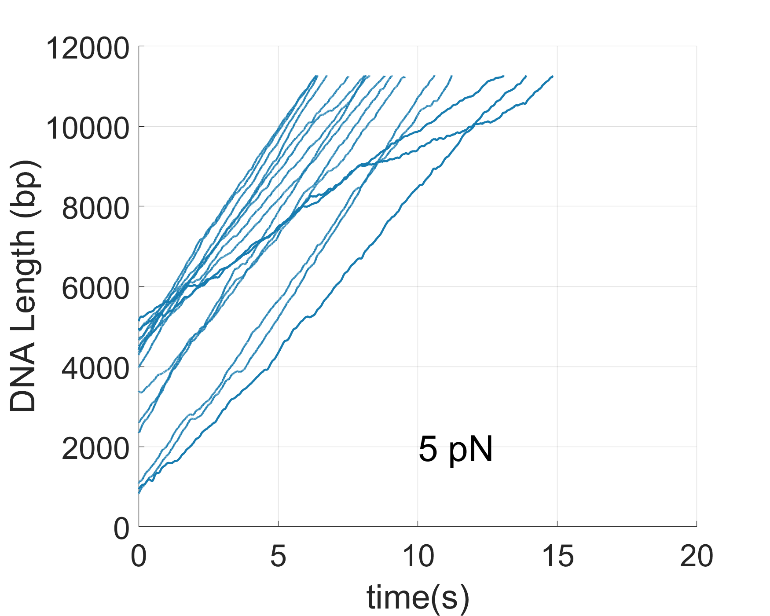


**Fig. S2. Measurements of length of DNA translocated versus time with the control phage DNA construct.** Left plot are measurements with 5 pN applied force and right plot are measurements with 30 pN applied force.


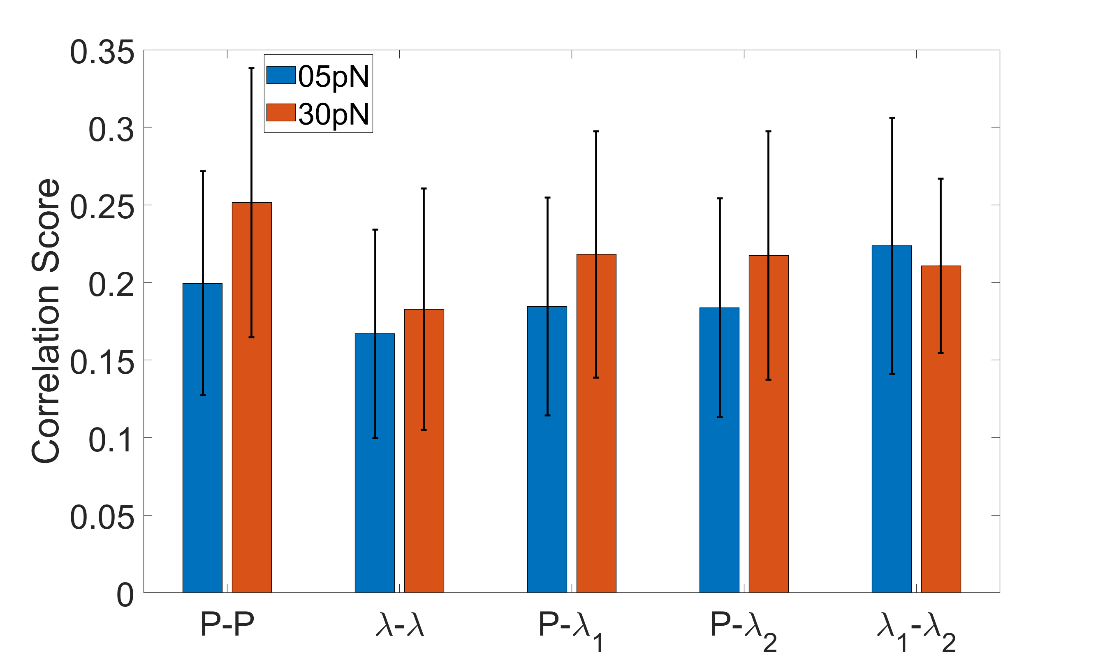


**Fig. S3. Additional packaging rate correlation analyses.** These analyses were performed in the same manner as described for Fig. 5B, except that a 200 bp sliding window was used instead of a 500 bp window (see methods).


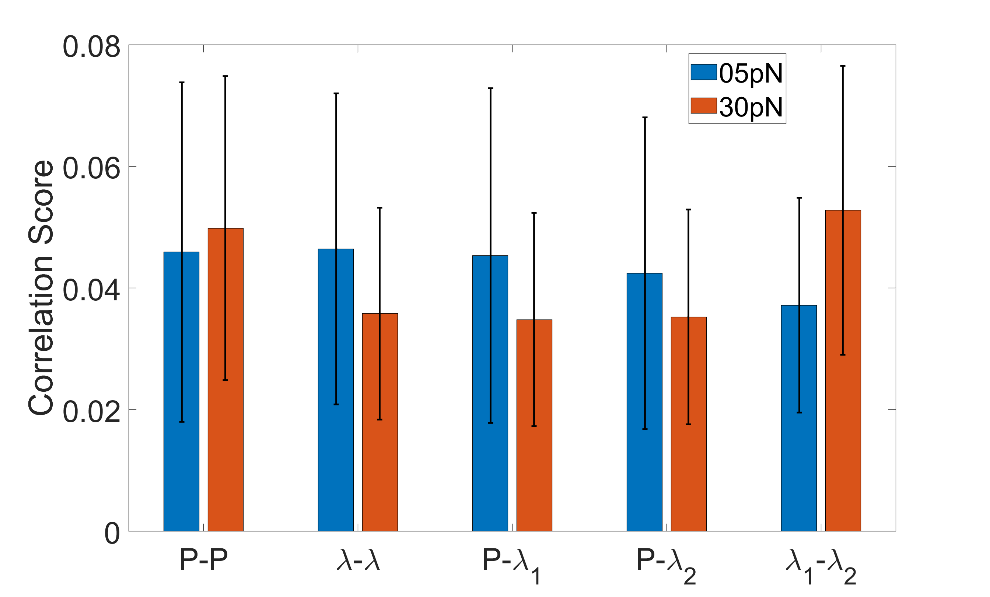

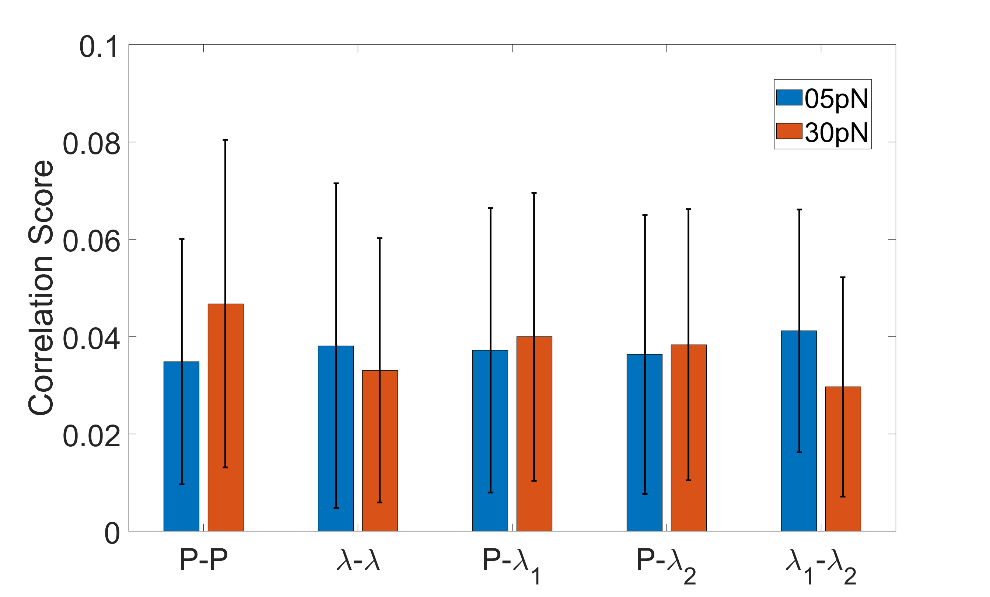


**Fig. S4. Additional pausing and slipping correlation analyses.** These analyses, for (A) pausing and (B) slipping, were performed in the same manner as described for Fig. 5C and 5D, except that a 200 bp sliding window was used instead of a 500 bp window (see methods).

**B**

**A**


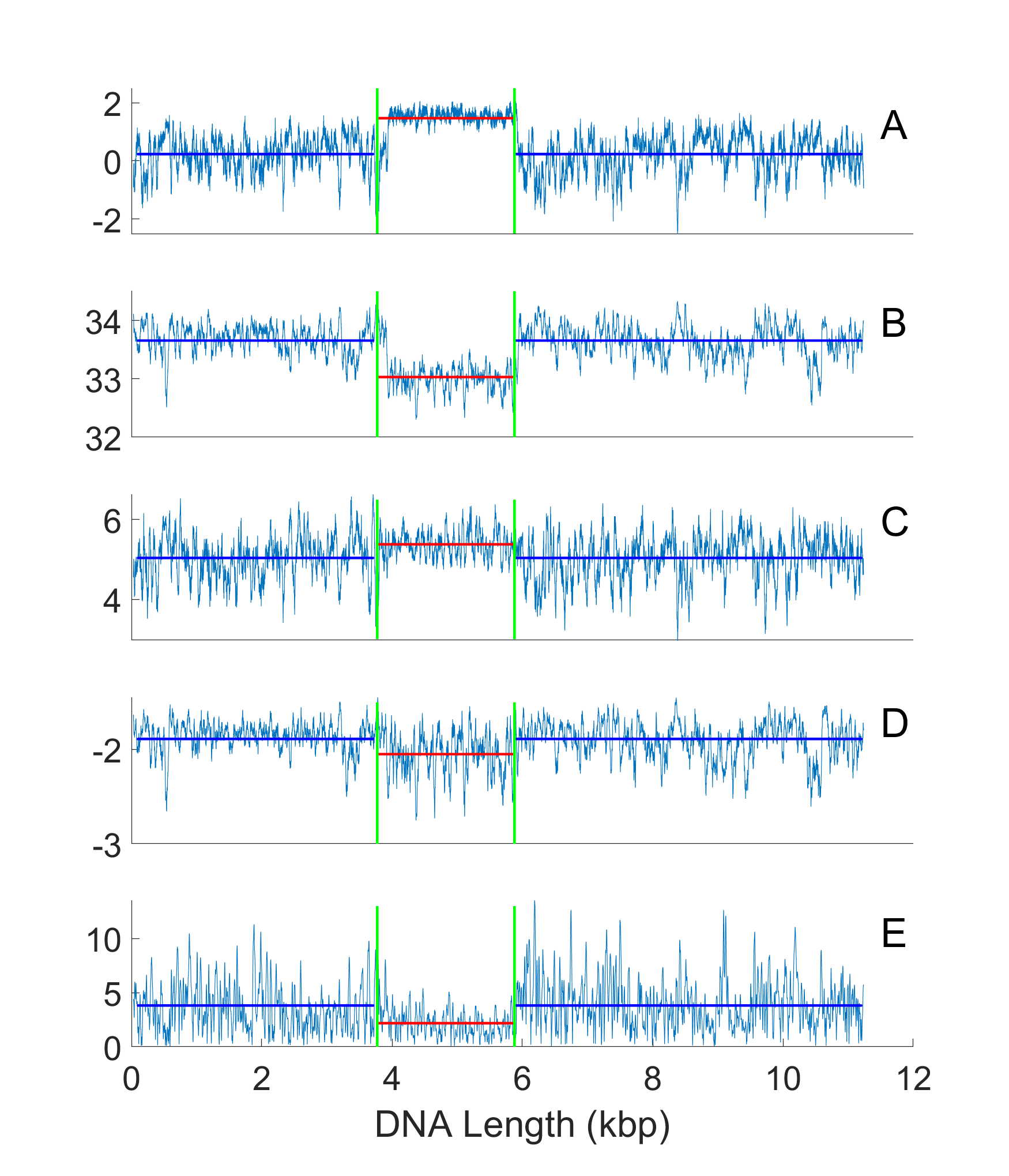


Twist angle

Roll angle

Curvature

ΔG (melting) melting

Bendability

**Fig. S5.** **Predicted sequence-dependent properties for the plasmid DNA construct containing the synthetic A-philic segment.** Predictions calculated using the “plot.it” and “bend.it” software packages using default settings (30 bp window size). All plots use the same horizontal axis at the bottom. Vertical green lines indicate the beginning and end of the synthetic A-philic segment. Horizontal red lines indicate the average property values for the A-philic section and horizontal blue lines indicate average property values for the flanking plasmid segments. (A) Roll angle (degrees; from conformational energy calculations). (B) Twist angle (degrees per helical turn; from conformational energy calculations). (C) Bendability (degrees; from DNAse I digestion experiments). (D) Free energy (ΔG) of melting (kcal/mol; from calorimetric studies). (E) Intrinsic curvature (degrees; consensus scale from DNAse I and nucleosome positioning data).

**Table S1.** **Mean predicted properties for the plasmid DNA construct containing the synthetic A-philic segment.** Means and standard errors in the means (SEM) for the properties plotted in Fig. S5. “Normal” refers to values for the flanking plasmid segments. “Difference” refers to the percent difference between the means for the A-philic and Normal sections.

| Parameter | A-philic Mean | A-philic  SEM | Normal Mean | Normal  SEM | Difference |
| --- | --- | --- | --- | --- | --- |
| (A) Roll | 1.47 | 0.048 | 0.23 | 0.035 | 539% |
| (B) Twist | 33.03 | 0.034 | 33.65 | 0.015 | -2% |
| (C) Bendability | 5.38 | 0.039 | 5.04 | 0.030 | 7% |
| (D) ΔG melt | -2.05 | 0.027 | -1.89 | 0.010 | 8% |
| (E) Curvature | 2.18 | 0.150 | 3.81 | 0.124 | -43% |
